# The catalytic mechanism of cyclic GMP‐AMP synthase (cGAS) and implications for innate immunity and inhibition

**DOI:** 10.1002/pro.3304

**Published:** 2017-10-25

**Authors:** Justin Hall, Erik C. Ralph, Suman Shanker, Hong Wang, Laura J. Byrnes, Reto Horst, Jimson Wong, Amy Brault, Darren Dumlao, James F. Smith, Leslie A. Dakin, Daniel C. Schmitt, John Trujillo, Fabien Vincent, Matt Griffor, Ann E. Aulabaugh

**Affiliations:** ^1^ Worldwide Medicinal Chemistry, Pfizer, Eastern Point Road Groton Connecticut 06340; ^2^ Hit Discovery and Lead Profiling, Pfizer Centers for Therapeutic Innovation (CTI), Pfizer, Eastern Point Road Groton Connecticut 06340; ^3^ Worldwide Medicinal Chemistry, Pfizer, 610 Main St Cambridge Massachusetts 02139

**Keywords:** cGAS, STING, 2′,3′‐cGAMP, cGAMP, OAS1, innate immunity

## Abstract

Cyclic GMP‐AMP synthase (cGAS) is activated by ds‐DNA binding to produce the secondary messenger 2′,3′‐cGAMP. cGAS is an important control point in the innate immune response; dysregulation of the cGAS pathway is linked to autoimmune diseases while targeted stimulation may be of benefit in immunoncology. We report here the structure of cGAS with dinucleotides and small molecule inhibitors, and kinetic studies of the cGAS mechanism. Our structural work supports the understanding of how ds‐DNA activates cGAS, suggesting a site for small molecule binders that may cause cGAS activation at physiological ATP concentrations, and an apparent hotspot for inhibitor binding. Mechanistic studies of cGAS provide the first kinetic constants for 2′,3′‐cGAMP formation, and interestingly, describe a catalytic mechanism where 2′,3′‐cGAMP may be a minor product of cGAS compared with linear nucleotides.

## Abstract 2

PDB Code(s): 5VDQ,; 5VDO,; 5VDP,; 5VDT,; 5VDR,; 5VDS,; 5VDW,; 5VDU,; 5VDV

## Introduction

Cyclic GMP‐AMP synthase (cGAS)* is a sensor of cytosolic double‐stranded DNA (ds‐DNA). ds‐DNA in the cytosol may occur from infection or mitochondrial damage. Metazoans have developed the ability to sense ds‐DNA in the cytosol as a trigger for the innate immune response.^1^, ^2^, ^3^ Although interferon and cytokine signaling are warranted to combat infectious ds‐DNA contaminants, recent studies have found that self‐ds‐DNA may occur in persisting autoimmune disorders such as systemic lupus erythematosus, suggesting inappropriate ds‐DNA sensing may be a contributor to autoimmune disease.^4^, ^5^, ^6^, ^7^

cGAS is activated by ds‐DNA binding to catalyze the cyclization of ATP and GTP to form a cyclic dinucleotide with mixed 2′,5′‐ and 3′,5′‐phosphodiester linkage (2′,3′‐cGAMP), which in turn activates stimulator of type 1 interferon genes (STING).^8^, ^9^, ^10^ Activated STING causes the activation of TBK1, which phosphorylates IRF3 allowing it to translocate to the nucleus where it triggers interferon‐inducible gene activation and interferon production.^8^, ^11^, ^12^, ^13^ Interestingly, intentional activation of STING through intratumoral injections of STING agonists has demonstrated antitumor properties and immunological memory, suggesting that while cGAS inhibition may benefit autoimmune patients, cGAS activation may be of therapeutic benefit in oncology.^14^, ^15^, ^16^

The relationship of cGAS and STING is both old (as much as much as 500 million years of co‐evolution),^17^ and interesting in that cGAS is a low‐activity enzyme while STING is a particularly avid binder of the cGAS product (*K*_d_ ∼4 n*M*).^18^ It has also been noticed by multiple investigators that cGAS produces linear homo‐ and hetero‐dinucleotides, including the unusual 2′,5′‐phosphodiester linked products GMP‐2′‐GTP and AMP‐2′‐GTP.^19^, ^20^ GMP‐2′‐GTP is thought to be a side reaction while AMP‐2′‐GTP is presumed to be the catalytic intermediate required for 2′,3′‐cGAMP production; if true this is a striking phenomenon as catalytic intermediates are seldom abundantly produced under physiological conditions, yet that appears to be the case for AMP‐2′‐GTP. OAS1 is a paralog of cGAS, it produces 2′,5′‐oligoadenylate as a secondary messenger during ds‐RNA‐induced innate immunity^21^; similarly, the 2′,5′‐phosphodiester link in GMP‐2′‐GTP and AMP‐2′‐GTP may distinguish them for as yet unrecognized roles. To better understand the role of cGAS and STING we undertook a study of the cGAS enzymatic mechanism and its production of linear and cyclic dinucleotides. We report here a novel SPR‐based kinetic analysis of cGAS, which in combination with HPLC, MS, and NMR assays suggest the majority of the cGAS enzymatic process is a futile cycle in terms of STING activation. We also present multiple structures of cGAS bound to dinucleotides and small molecule inhibitors that allows us to expand on the existing theory for cGAS activation and propose targeted sites for inhibitor or activator binding.

## Results

### Interaction with Asp_227_ causes catalytic acid alignment

**Figure 1:**
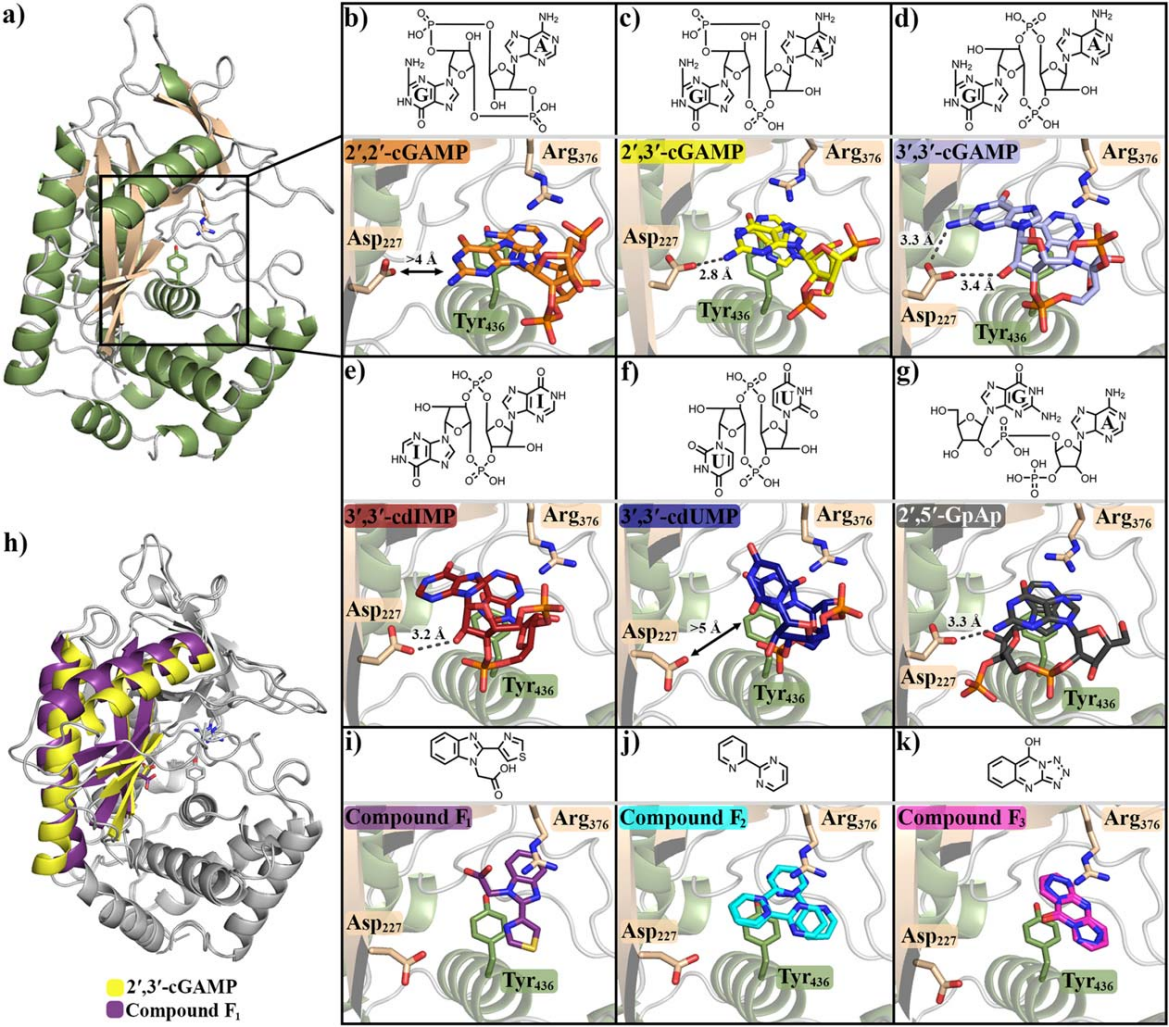
Tyr_436_ and Arg_376_ form a binding site for aromatic rings. (a) cGAS_161_ bound to (b) 2′,2′‐cGAMP, (c) 2′,3′‐cGAMP, (d) 3′,3′‐cGAMP, (e) 3′,3′‐cdIMP, (f) 3′,3′‐cdUMP, and (g) 2′,5′‐GpAp. h) Changes between the inactive β‐sheet pose (compound F_1_ bound) and β‐pseudo active conformations (2′,3′‐cGAMP bound). cGAS_161_ bound to compound (i) F_1_, (j) F_2_, and (k) F_3_. Compounds structures are shown above protein structures; F_2_ and F_3_ are modeled with more than a single pose due to uncertainty in their electron density. Figure generated using Pymol with pdb structures 5VDO (cGAS_161_•2′,2′‐cGAMP), 5VDP (cGAS_161_•2′,3′‐cGAMP), 5VDT (cGAS_161_•3′,3′‐cGAMP), 5VDR (cGAS_161_•3′,3′‐cdIMP), 5VDS (cGAS_161_•3′,3′‐cdUMP), 5VDQ (cGAS_161_•2′,5′‐GpAp), 5VDW (cGAS_161_•F_1_), 5VDU (cGAS_161_•F_2_), and 5VDV (cGAS_161_•F_3_)^28^, ^33^, ^34^

ds‐DNA binding causes two major changes to the apo cGAS secondary structure. The first is residues Gly_207_‐Val_218_ (*Homo sapiens* numbering for changes seen in *Mus musculus* (PDB 4O6A)^22^ and *Sus scrofa* (PDB 4KB6))^23^ change from disordered to a regular secondary structure (β‐strand between Gly_207_‐Asn_210_, α‐helix between Gly_212_‐Val_218_), and the second is a ∼1 Å shift of the β‐sheets containing the catalytic acids (Glu_225_, Asp_227_, and Asp_319_) towards the active site (Fig. 1). In the absence of ds‐DNA, human cGAS can adopt a cyclic dinucleotide‐dependent structure similar to the second of these structural changes, where the catalytic acid containing β‐sheets have moved towards the active site (see PDB 4O67 and 4O69)^22^ while residues Gly_207_‐Val_218_ remain disordered. Since only the shift in the β‐sheets has occurred in this dinucleotide‐dependent structural change, we shall distinguish this conformation from the fully active form, referring to it as “β‐pseudo‐active” for the changes in the β‐sheets. To study the β‐pseudo‐active form we obtained structures of an N‐terminal truncation of cGAS starting at residue 161 (cGAS_161_)^22^ in complex with five cyclic dinucleotides (2′,2′‐cGAMP, 2′,3′‐cGAMP, 3′,3′‐cGAMP, 3′,3′‐cdIMP and 3′,3′‐cdUMP), and the linear 2′,5′‐GpAp dinucleotide (Supporting Information Fig. S1 and Supporting Information Tables S1 and S2). In four of these structures, cGAS assumes the β‐pseudo‐active conformation (Table 1).

**Table 1:** Cyclic Dinucleotide Affinities and Conformation States for cGAS

	K_d_ (μ*M*)		
Ligand	apo cGAS_161_	ds‐DNA•cGAS_161_	cGAS Conformation
2′,2′ cGAMP	>500	>500	Inactive
2′,3′ cGAMP	89 ± 6^a^	56 ± 1	β‐pseudo‐active
3′,3′ cGAMP	21 ± 5	8.9 ± 0.3	β‐pseudo‐active
3′,3′ cdIMP	>500	>500	β‐pseudo‐active
2′‐5′GpAp	215 ± 81	ND^b^	β‐pseudo‐active
3′,3′ cdUMP	>500	>500	α‐pseudo‐active

a Data are the average and standard deviation of two or more SPR experiments.

b Data were not determined (ND).

There is always an interaction between the catalytic acid Asp_227_ and the dinucleotide when the β‐pseudo‐active conformation occurs. In most cases Asp_227_ forms a hydrogen bond with the amino group of the guanine base with an approach between 2.5 and 3.3 Å (Fig. 1). The exception to this is 3′,3′‐cdIMP which does not have an amino group on its base, but does interact with Asp_227_ through the 2′‐OH guanine ribose (3.2 Å).

There is no interaction between Asp_227_ and 3′,3′‐cdUMP or 2′,2′‐cGAMP; in these structures cGAS retains an inactive pose. For 3′,3′‐cdUMP there is no amino group on its base, and the smaller pyrimidine does not penetrate as deeply into the active site as purines. 2′,2′‐cGAMP contains an amino group on its guanine base; however, 2′,2′‐cGAMP is considerably displaced from the active site compared with 2′,3′‐cGAMP or 3′,3′‐cGAMP (Fig. 1) with its amino group interacting with Asp_319_ (2.9 Å) instead of Asp_227_.

Most of these dinucleotides have affinities weaker than 500 μ*M* (the top concentration tested). 2′,3′‐cGAMP and 3′,3′‐cGAMP are more tightly bound than the other dinucleotides, and we were able to detect a modest (∼2‐fold) increase in affinity for both between apo cGAS_161_ and ds‐DNA‐bound cGAS (Table 1, Supporting Information Fig. S2). The change in affinity is consistent with the preordering of Asp_227_ by ds‐DNA.

### The substrate orientation is thermodynamically preferred for 2′,3′‐cGAMP

Structures of ds‐DNA bound to cGAS shows ds‐DNA packs against residues between Gly_207_‐Val_218_, inducing their active conformation.^22^, ^23^ In general, Gly_207_‐Val_218_ do not adopt a regular secondary structure without ds‐DNA, though strong electron density can be seen for these residues with some ligands. In our 2′,3′‐cGAMP structure, the electron density is sufficient to model Gly_207_‐Val_218_. Comparison to the existing human cGAS 2′,3′‐cGAMP‐bound structure (PDB 4O67) shows good agreement, the only exception is for the modeling of residues Ile_220_ and Ser_221_. In our structure we observe a close contact between Lys_219_ and Ala_222_ that could be modeled as a continuation of the main chain into a β‐turn, which is the case for 4O67; however, electron density of side chains in our structure make it clear there is actually a break in the main chain and not a β‐turn. 4O67 is the only structure of cGAS from any organism that models a β‐turn at Ile_220_ and Ser_221_ (Supporting Information Fig. S3).

Our structure, 4O67, and a *M. musculus* structure with both ds‐DNA and 2′,3′‐cGAMP (PDB 4K9B)^19^ all model 2′,3′‐cGAMP with the adenine base above Tyr_436_, and the guanine base near Leu_209_. This is the same position seen for the adenine and guanine base in the structure of substrate‐bound cGAS (PDB 4KB6), we therefore refer to this base orientation for 2′,3′‐cGAMP as being in the “substrate orientation”.

A second ds‐DNA‐ and 2′,3′‐cGAMP‐bound *M. musculus* structure (PDB 4LEZ)^24^ has the guanine and adenine bases switched relative to the positions in our model, 4O67, and 4K9B. The discrepancy between 4LEZ and other models may be due to how 2′,3′‐cGAMP was introduced to the 4LEZ crystals. In our and the 4O67 structure, 2′,3′‐cGAMP was added to apo enzyme, while in 4LEZ, 2′,3′‐cGAMP was generated enzymatically in the crystal from ATP and GTP. Since the AMP‐2′‐GTP intermediate must switch base positions during cyclization, there is the interesting possibility that the electron density captured in the 4LEZ structure represents a mixture of reaction intermediate and final product. Thus, it would appear that the pose of 2′,3′‐cGAMP with adenine above Tyr_436_ is the thermodynamically favored position occurring after product rebinding.

### Tyr_436_ and Arg_376_ form a binding site for aromatic rings

A screen of the Pfizer fragment chemical library discovered several binders of cGAS. Fragment screens are run to find low affinity chemical leads, which are subsequently developed into more potent drug‐like inhibitors. All the fragments described here bind at the active site and are weak inhibitors, the development of one of our fragment hits from a weak (∼200 μ*M*) to a potent (∼200 n*M*) inhibitor is described elsewhere.^25^ We detail here three fragments hits for their insights into the cGAS activation mechanism and direct interested readers to our other publication for the development of a fragment hit to a potent inhibitor of cGAS.^25^

Each of the fragment binders shown here (F_1_–F_3_) are small (∼150–250 Da) with weak affinity (∼100–300 μ*M*) (Supporting Information Table S3 and Supporting Information Fig. S2). The Fo‐Fc maps for F_2_ and F_3_ are not sufficient to unambiguously define atomic positions, which seems to be related to multiple modes of binding and internal pseudo‐symmetry. Indeed, compared with the dinucleotide structures which have specific interactions that define their positions shows far less ambiguity in unbiased Fo‐Fc maps, despite these dinucleotides being generally weaker binders than the fragments and at comparable resolutions (Fig. 1 and Supporting Information Fig. S1).

Each compound binds to the same site formed between the side chains of Tyr_436_ and Arg_376_ (Fig. 1). This site is occupied by the adenine base in structures of ATP, 2′,2′‐cGAMP, 2′,3′‐cGAMP, or 3′,3′‐cGAMP, and is composed primarily of London dispersion interactions between the ring system of these binders with the phenyl ring of Try_436_. These interactions suggest this site could accommodate most 2‐ or 3‐ring aromatic systems, which is consistent with this site needing to bind both the adenine and guanine bases during catalysis. Additionally, we observe that in all our cyclic dinucleotides structures, this site had better defined electron density than the other nucleobase site, even for identical ring systems like 3′,3′‐cdIMP and 3′,3′‐cdUMP. The binding site formed by Tyr_436_ and Arg_376_ is therefore a site of binding with broad specificity for aromatic rings, be they nucleobases or small molecule fragments.

### cGAS can form an α‐helix at Gly_212_‐Val_218_ in the absence of ds‐DNA

Compounds F_1_–F_3_ bind distant (∼10 Å) from Asp_227_ and do not elicit the β‐pseudo‐active conformation. For compounds F_1_ and F_2_, one chain of the asymmetric unit (chain A) has strong electron density for Gly_207_‐Val_218_, which adopts a short β‐strand at Gly_207_‐Asn_210_, and an α‐helix at Gly_212_‐Val_218_ while the other chain in the asymmetric unit does not. Compound F_3_ has the same conformation but weaker density for these residues. Interestingly, while 3′,3′‐cdUMP does not cause a β‐pseudo‐active conformation, one molecule of the asymmetric unit (chain B) adopts the same conformation for Gly_207_‐Val_218_ seen for compounds F_1_–F_3_. Thr_211_ of the short β‐strand is important for coordinating the nucleobase and determining the specificity of the 2′ or 3′‐OH bond formation (20), while Ser_213_ of the α‐helix may bind phosphates of the linear intermediate (see PDB 4K99 and 4K9A).^19^ These structural changes are therefore critical for cGAS activity.

The formation of the short β‐strand and α‐helix occur in the ds‐DNA‐bound structures of cGAS, yet there are no molecular contacts to explain their presence in these structures. Thus, these data seem to describe a naturally occurring propensity for this conformation, which is enriched in one of the two chains in the asymmetric unit of these crystals.

**Figure 2:**
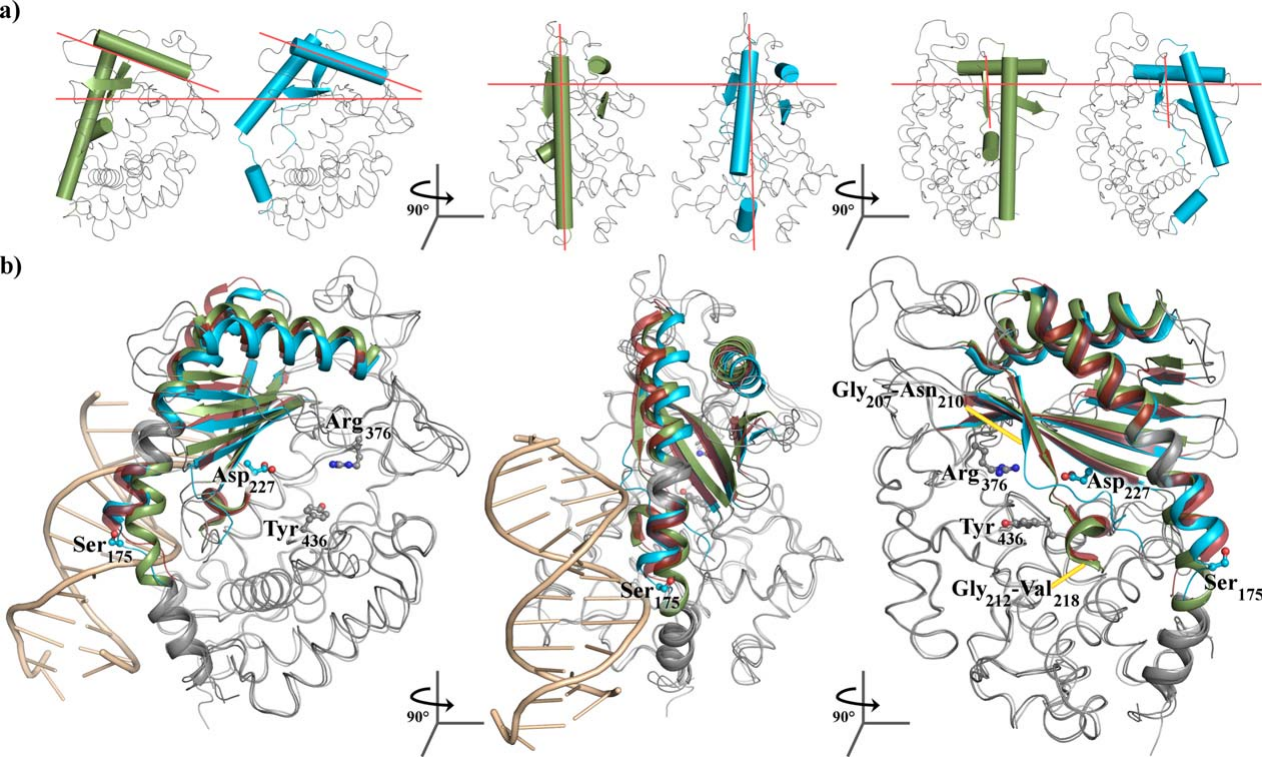
cGAS is able to assume pseudo‐active states without binding to ds‐DNA. (a) cGAS in the α‐pseudo‐active (green) or β‐pseudo‐active conformation (blue). Orange lines are a guide for the eye corresponding to the approximate orientation of these same secondary structures in the ds‐DNA bound cGAS structure. (b) Overlay of cGAS bound to F_1_ (green) showing the α‐pseudo‐active conformation, 2′,3′‐cGAMP showing the β‐pseudo‐active conformation (blue), or ds‐DNA (red) showing the fully active conformation. In the third panel the ds‐DNA is forefront and has been removed for clarity. Images were generated using Pymol with pdb structures 5VDP (2′,3′‐cGAMP‐bound cGAS_161_), 5VDW (F_1_‐bound cGAS_161_), and 4KB6 (ds‐DNA‐, ATP‐, and GTP‐bound *S. scrofa* cGAS)

These structures demonstrate the ability of cGAS to form an active‐like conformation for Gly_207_‐Val_218_ while the catalytic acid containing β‐sheets remain in the inactive pose. This is the opposite effect seen for the β‐pseudo‐active conformation. We call this second pseudo‐active conformation “α‐pseudo‐active” in reference to the α‐helix at residues Gly_212_‐Val_218_. That distinct α‐ and β‐pseudo‐active states exist suggests these two conformations are either independent or mutually exclusive, requiring DNA‐binding to coordinate both conformation transitions (Fig. 2).

### An SPR‐based enzymatic assay to determine kinetic constants

**Figure 3:**
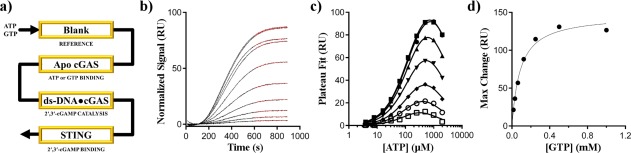
An SPR‐based enzymatic assay. (a) Schematic for SPR sensor layout. Individual channels are indicated by yellow boxes and the flow‐path is indicated by the black arrow. b) Representative data for normalized STING_155–341_ response over 900 s injections; data are for a fixed concentration of GTP (0.5 m*M*), variable ATP (3.9 μ*M* to 2 m*M* over 10 points). The red lines show the fit of the data to a single exponential association from 600 to 900 s to determine the RU maxima. (c) Extrapolated RU maxima for seven fixed GTP concentration (16 μ*M* to 1 m*M*) over variable ATP concentrations (3.9 μ*M* to 2 m*M*). Solid lines show the fit of the data to equation (2) using a GTP‐concentration independent *K*_M.ATP_ value with substrate inhibition observed at high nucleotide concentrations. (d) Calculated maximum response values from panel c were plotted as a function of the GTP concentration. The black line shows the fit of the data using the Michaelis‐Menten equation. Error bars of the fits for are within the size of the data markers in panels (c) and (d)

The Biacore T200 SPR microfluidics were designed with a serpentine flow; samples injected must pass along all four flow cells during data collection. We reasoned that if we immobilized apo cGAS, ds‐DNA bound cGAS, and STING_155–341_ in series we could inject ATP and GTP, detect binding to apo cGAS, form 2′,3′‐cGAMP with ds‐DNA bound cGAS, and finally detect 2′,3′‐cGAMP using STING_155–341_ as a sensor [Fig. 3(a)].

*K*_M_ values reflect the steady‐state equilibrium between free enzyme and all other enzyme species. In theory, *K*_M_ values for DNA‐bound cGAS_161_ could be determined by directly monitoring the SPR response of this channel as a function of ATP and GTP. However, the ds‐DNA bound cGAS_161_ channel had a near‐zero response resonance unit (RU) signal for all concentrations of ATP and GTP which made a direct *K*_M_ calculation from the cGAS channel impossible. Monitoring the STING_155–341_ response showed 2′,3′‐cGAMP was being produced by ds‐DNA bound cGAS; thus we used the STING response to determine *K*_M_ in lack of a direct cGAS response. Though STING was necessary for monitoring substrate turn over in this system, we suspect direct analysis of the change in RU of the enzyme channel should work for other systems.

Although binding is generally seen as a positive increase in SPR response, it has been observed that for some proteins a negative signal can occur which is attributed to conformation changes associated with binding.^26^ The ds‐DNA bound cGAS response appears to report the summation of positive (mass accumulation) and negative (conformation changes) responses occurring during catalysis. Since mass accumulation and conformational changes should be distinct with distinct enzymes, we suspect this zero‐sum phenomenon will not occur for other enzymes.

We have determined there are criteria that must be met for SPR to be used for activity assays (see “Discussion” section). One of the most important is that the sensor protein must bind and give a measurable response for the desired analyte while being insensitive to other chemical matter present in the assay samples (see below). STING binds 2′,3′‐cGAMP with n*M* affinity (4 n*M* reported K_d_),^18^ and gave a clear positive response when titrated with commercial 2′,3′‐cGAMP standards. No response was observed when ATP or GTP was injected in the absence of the other nucleotide indicating STING_155–341_ does not bind ATP or GTP at the concentrations used here.

**Figure 4:**
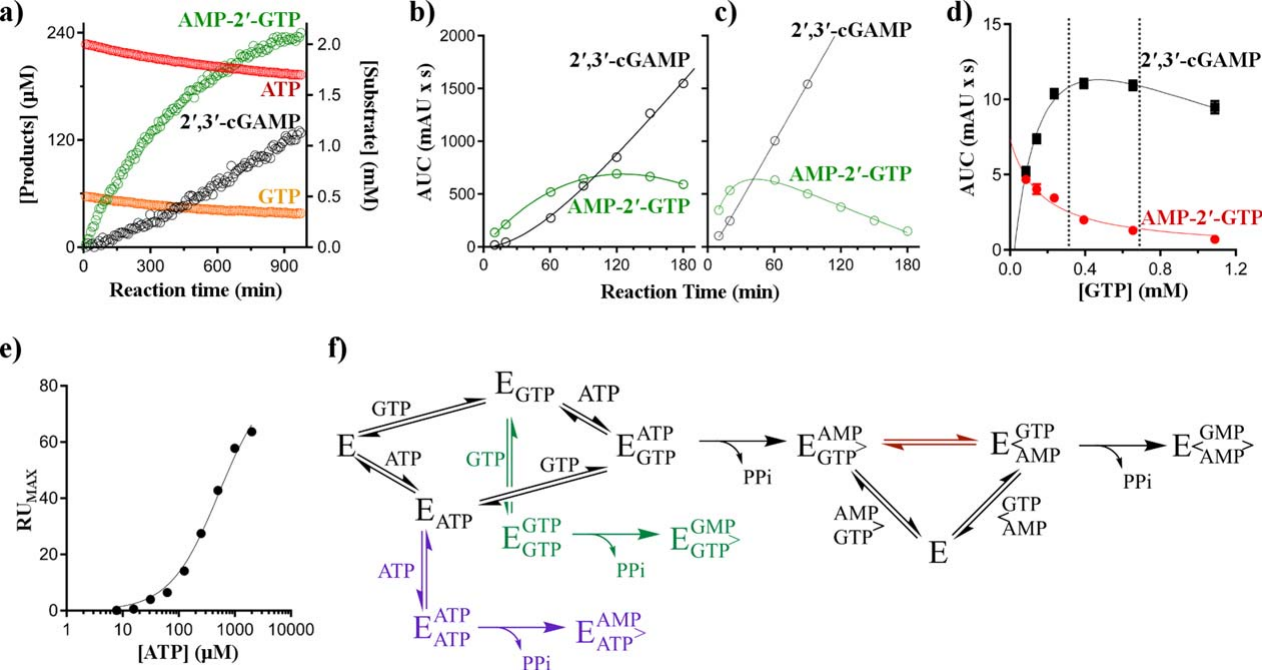
Linear homo‐ and hetero‐dinucleotides are the major products of cGAS. (a) NMR‐based full length cGAS reaction progress monitoring the loss of substrates (ATP and GTP, red and orange) and accumulation of AMP‐2′‐GTP (green) and 2′,3′‐cGAMP (black). HPLC‐based monitoring of AMP‐2′‐GTP (green) and 2′,3′‐cGAMP (black) from either b) cGAS_161_ or (c) full length cGAS. (d) Steady‐state rates for full length cGAS formation of 2′,3′‐cGAMP (black) or AMP‐3′‐ATP (red) at 1.1 m*M* ATP as a function of GTP; marked region represents approximate cellular GTP concentrations. (e) Maximum RU signal for STING binding to 2′,3′‐cGAMP at increasing concentrations of a fixed ratio (2:1) of ATP and GTP. The concentration of GTP is omitted on the x‐axis for simplicity. (f) Mechanism for the formation of homo‐ and hetero‐dinucleotides by cGAS (E); cGAS is shown after ds‐DNA activation; AMP‐2′‐GTP release or reorganization on‐enzyme (red path) are illustrated; schematic based on mechanism proposed by Gao et al^19^

AMP‐2′‐GTP has been previously observed in cGAS reactions and was an important clue to seminal studies on the cGAS enzymatic mechanism [Fig. 4(f)]^19^ where it was described as a pathway intermediate. To determine if STING_155–341_ binds the intermediate, we used full length cGAS to prepare four reaction mixtures with variable concentrations of 2′,3′‐cGAMP and AMP‐2′‐GTP. Samples were tested in SPR and the 2′,3′‐cGAMP concentration was determined from the STING response compared with 2′,3′‐cGAMP standards. The total SPR response on the STING channel was in close agreement to the expected result for total 2′,3′‐cGAMP as measured by NMR, with no additional response observed from AMP‐2′‐GTP (Table 2), even when intermediate was present at 10‐fold excess over 2′,3′‐cGAMP. The simplest explanation for these data is AMP‐2′‐GTP does not bind to STING_155–341_ at the top concentrations tested here (∼50 μ*M*). We therefore concluded that STING can be used as a selective sensor for 2′,3′‐cGAMP without interference from ATP, GTP or AMP‐2′‐GTP.

**Table 2:** SPR Response to Mixtures of AMP‐2′‐GTP and 2′,3′ cGAMP Show No Binding to STING for AMP‐2′‐GTP

		Reaction Conditions [ATP:GTP] (μ*M*)			
	Product	250:500	500:1000	1000:2000	2000:4000
NMR	[AMP‐2′‐GTP] (μ*M*)	31 ± 10^a^	92 ± 31	184 ± 61	216 ± 72
	[2′,3′ cGAMP] (μ*M*)	20 ± 7	38 ± 13	44 ± 15	24 ± 8
SPR	[2′,3′ cGAMP] (μ*M*)	10 ± 3	25 ± 8	31 ± 10	12 ± 4

a Values ± error were determined from NMR peak integration (top) or SPR RU signal change (bottom) compared with 2′,3′ cGAMP controls.

*K*_M_ values for 2′,3′‐cGAMP production were determined using SPR as well as HPLC, MS, and NMR assays. Values compiled in Table 3 show good agreement between techniques, and between both full length cGAS and cGAS_161_.

**Table 3:** *cGAS Steady‐State Apparent* K*_M_ Values* a

*K*_M.GTP_ (μ*M*)	*K*_IS.GTP_ (μ*M*)^b^	*K*_M.ATP_ (μ*M*)	*K*_IS.ATP_ (μ*M*)^b^	Enzyme	Method
90 ± 12	ND^c^	140 ± 4	2700 ± 100	cGAS_161_	SPR
90 ± 20	ND	190 ± 20	2500 ± 500	cGAS_161_	SPR
190 ± 50	ND	120 ± 30	ND	Full length	NMR
160 ± 50^d^	1500 ± 500^e^	ND	ND	Full length	HPLC
40 ± 10	1500 ± 600	50 ± 10	700 ± 200	Full length	MS
22 ± 2	1700 ± 200	24 ± 1	3600 ± 500	Full length	MS

a *K*_M_ values are apparent *K*_M_ values with errors of fit (see “Materials and methods” Section).

b As presented in the results, *K*_IS_ values were dependent upon the concentration of the non‐varied nucleotide. Presented values were determined at about 1 m*M* ATP or 0.3 m*M* GTP.

c Data were not determined (ND).

d Value is the GTP IC_50_ value for inhibition of AMP‐3′‐ATP formation.

e Value was determined from equation (2) using a set *K*_M.GTP_ value of 160 μ*M*.

Using the SPR method, the apparent ATP *K*_M_ value was determined using a range of GTP concentrations. Using an extra sum‐of‐squares F‐test, a GTP concentration‐independent *K*_M.ATP_ value was supported over a GTP dependent *K*_M_ value (*P* = 0.18), suggesting a lack of cooperativity between ATP and GTP binding. Similarly, ATP binding to apo cGAS_161_ showed no dependence on the concentration of GTP. Similar *K*_d_ and *K*_M_ values for ATP (235 ± 97 and 190 ± 20 μ*M*) are consistent with a subsequent step occurring at a rate slower than the rate of substrate association/dissociation (e.g. slow conformational changes or chemistry). We observed no RU signal for GTP binding to apo cGAS_161_ (up to 1 m*M* GTP), including conditions where ATP was pre‐bound to cGAS_161_ (up to 2 m*M* ATP). The simplest explanation of these data is that apo cGAS_161_ does not bind GTP tightly; however, the absolute RU change for ATP was muted compared with non‐substrate analytes [Supporting Information Fig. S2(E)]. We therefore cannot rule out that GTP binding results in a conformation change and a net‐zero RU signal.

### Substrate inhibition occurs from competitive side reactions

ATP titrations show a clear substrate inhibition pattern for both cGAS_161_ in SPR, and full length cGAS in HPLC and MS assays. Using SPR, the apparent K_IS_ value increased with increasing GTP concentrations. However, no substrate inhibition was seen at up to 2 m*M* ATP when the ratio of ATP and GTP was constant, suggesting substrate competition occurs between ATP and GTP.

The apparent substrate inhibition is most simply explained by the two nucleotides competing with each other to form AMP‐3′‐ATP or GMP‐2′‐GTP instead of 2′,3′‐cGAMP. In agreement with this, cGAS has been observed to produce both AMP‐3′‐ATP and GMP‐2′‐GTP (Supporting Information Fig. S4).^19^, ^20^ We found GTP behaved as a competitive inhibitor of AMP‐3′‐ATP formation using cGAS_161_ or full length cGAS in HPLC titrations. This substrate inhibition is the result of a random‐ordered, bi‐substrate reaction, where one reactant can compete with the second for binding. This also explains the GTP‐concentration dependence of the ATP K_IS_ values. As mentioned earlier, when ATP and GTP are kept at a constant molar ratio, no substrate inhibition was observed because the ratio of 2′,3′‐cGAMP to homo‐dinucleotides would also remain constant. The apparent *K*_M_ for AMP‐3′‐ATP formation in the absence of GTP was 3700 ± 1200 μ*M*.

### Linear homo‐ and hetero‐dinucleotides are the major initial products of cGAS

Though we first identified the formation of AMP‐3′‐ATP and GMP‐2′‐GTP in HPLC and MS by running control reactions with single nucleotide substrates, we could also observe both products in MS experiments under physiologically relevant concentrations of ATP and GTP (2 m*M* and 0.5 m*M*)^27^ with cGAS_161_ or full length cGAS. Exact dinucleotide concentrations were not possible to determine lacking MS ionization controls, but peak intensities suggests the homo‐linear products are ∼10% of the total reaction (Supporting Information Fig. S4). Similarly, the AMP‐3′‐ATP product could be detected at all non‐zero GTP concentrations tested using HPLC [Fig. 4(d)], and at physiological substrate concentrations,^27^ comprised *∼*20% of the product compared with 2′,3′‐cGAMP for full length cGAS. The GMP‐2′‐GTP peak was only observed in HPLC in the absence of ATP, suggesting this product is not as readily formed compared with AMP‐3′‐ATP.

In addition to the homo‐linear products, we observed the production of linear AMP‐2′‐GTP using continuous reaction monitoring of full length cGAS in NMR and reaction‐arrested MS and HPLC. In our studies, AMP‐2′‐GTP is produced in significant abundance initially, while 2′,3′‐cGAMP is not [Fig. 4(a–c)]; this result is independent of the cGAS form used in the study (i.e. cGAS_161_ or full length). These data strongly suggest a mechanism where the majority of substrate flows through AMP‐2′‐GTP, which is released to solution and then must rebind in competition with ATP and GTP to form 2′,3′‐cGAMP. Consistent with this mechanism, there is a lag in the initial rate of 2′,3′‐cGAMP formation, but no lag in the initial rate of AMP‐2′‐GTP formation at saturating ATP and GTP concentrations (Fig. 4).

### cGAS can produce 2′,3′‐cGAMP without releasing AMP‐2′‐GTP to solution

In a closed system (such as an NMR tube), AMP‐2′‐GTP released from the enzyme will accumulate until it reaches a concentration sufficient to compete with ATP and GTP for rebinding to free enzyme. Thus, end point activity assays may miss the difference in AMP‐2′‐GTP and 2′,3′‐cGAMP concentrations at early time points while continuous methods like NMR will not. In contrast to a closed tube, the continuous flow through the SPR system limits the concentration of AMP‐2′‐GTP that can accumulate according to the rate of production and the rate of flow through the cell. Thus, as ATP and GTP concentrations increase past their *K*_M_ in SPR they will competitively block AMP‐2′‐GTP rebinding to cGAS, decreasing 2′,3′‐cGAMP production. In effect this would look like substrate inhibition. To separate substrate inhibition of 2′,3′‐cGAMP production due to ATP and GTP competing with AMP‐2′‐GTP, from inhibition by side reactions (e.g. AMP‐3′‐ATP and GMP‐2′‐GTP formation), we used a fixed ratio of ATP to GTP with final concentrations up to 25‐fold their *K*_M_ and did not observe inhibition of 2′,3′‐cGAMP production [Fig. 4(e)]. These data are consistent with a catalytic mechanism where AMP‐2′‐GTP is not subject to ATP or GTP competition; this is most easily explained by a mechanism where AMP‐2′‐GTP is not released but instead reorients on the enzyme.

These SPR data appear to be in conflict with the NMR data which demonstrate a large accumulation of AMP‐2′‐GTP in solution. Reconciliation of the NMR and SPR data results in a mixed mechanism, where the majority of AMP‐2′‐GTP dissociates and can rebind after enough has accumulated to compete, but where a minor portion reorients directly on‐enzyme to form 2′,3′‐cGAMP in a process that is not competitive with ATP or GTP [Fig. 4(f), red arrows].

NMR experiments using full length cGAS and physiological concentrations of ATP and GTP (2 and 0.5 m*M*, 10‐fold their *K*_M_)^27^ show ∼250 μ*M* AMP‐2′‐GTP accumulates at steady‐state [Fig. 4(a)]. These data suggest either slow chemistry occurs for AMP‐2′‐GTP loss relative to formation, or the *K*_M_ of AMP‐2′‐GTP is around 25 μ*M*. Consistent with a weak *K*_M_, we did not observe binding for AMP‐2′‐GTP at a top concentration of 50 μ*M* using apo cGAS_161_ in SPR.

Despite the apparent weak affinity of AMP‐2′‐GTP, and its competition with ATP and GTP, this may not have a significant biological effect since STING is a tight binder of 2′,3′‐cGAMP (4 n*M* K_d_)^18^ requiring only a small amount to cause signaling. The SPR results demonstrate a mechanism exists where a fraction of AMP‐2′‐GTP is not subject to ATP or GTP competition, such as through reorientation on the enzyme. Based on the full length NMR steady‐state concentration of AMP‐2′‐GTP, a rough approximation suggests that if the fraction of AMP‐2′‐GTP that stays on‐enzyme is >0.1%, the on‐enzyme path should produce the requisite levels of 2′,3′‐cGAMP needed for STING sensing before enough AMP‐2′‐GTP accumulates to compete with ATP and GTP for enzyme rebinding. Thus AMP‐2′‐GTP appears to be both the major initial product of cGAS and its release is a futile cycle in terms of 2′,3′‐cGAMP production and STING activation.

## Discussion

During catalysis cGAS must accommodate a swap of adenine and guanine nucleobases in its active site. This is enabled by Tyr_436_ and Arg_376_, which create a binding site for disparate aromatic rings. Since this site is essential for binding of both substrates and intermediate, small molecules binding this site cause enzyme inhibition (Fig..1)^25^, ^28^

When analyzing the inactive‐to‐active transition of cGAS, we see there are α‐ and β‐pseudo‐active conformations that mimic the true ds‐DNA‐dependent active state. The α‐pseudo‐active state occurs without clear ligand provocation, suggesting it may be a regularly occurring state in the absence of ds‐DNA, whereas the β‐pseudo‐active state is observed when Asp_227_ is engaged.

In the absence of ds‐DNA, we only observe α‐ and β‐pseudo‐active states separately, suggesting these states are either independent or mutually exclusive. Our structural analysis supports a model where α‐ and β‐pseudo‐active states are mutually exclusive, which is consistent with a model for activation proposed by Civril et al.^23^ According to this model, when ds‐DNA binds, it breaks the long N‐terminal helix of cGAS into two daughter helices (α1 and α2) at Ser_175_ (Fig. 2). This break is observed in ds‐DNA‐bound structures where the positive dipole of the α2 helix interacts with the DNA backbone^19^, ^23^, but also occurs in the cyclic dinucleotide‐induced β‐pseudo‐active states. The potential importance of the long N‐terminal helix formed the basis for a Leu_174_Asn mutant by Civril et al. (in recognition of its importance they refer to this helix as the “spine” of cGAS) where they hypothesized the helical break allows Leu_174_ to stabilize the active form through the formation of an α‐helix at Gly_212_‐Val_218_. Strikingly, they showed Leu_174_Asn is able to bind DNA but no longer produces 2′,3′‐cGAMP, these results are consist with the idea that α‐ and β‐pseudo‐active confirmations are mutually exclusive. Similarly, others have found amino acid substitutions along this helix (e.g. Lys_173_Ala/Arg_176_Ala in *H. sapien*s or Arg_158_Ala in *M. musculus*) greatly reduce catalytic activity.^23^, ^24^

In the α‐pseudo‐active structures, the long N‐terminal helix is intact and the single‐turn α‐helix is formed at Gly_212_‐Val_218_. When ds‐DNA breaks the long N‐terminal helix, it also positions the C‐terminal end of the daughter helix α2 towards ds‐DNA, aligning Gly_207_‐Asn_210_ to form a short β‐strand with Val_228_‐Lys_231_. The importance of the short β‐strand for cGAS activity has been described by others, who liken it to the “activation loop” of kinases^22^ Though ds‐DNA binding may also help form the α‐helix at Gly_212_‐Val_218_ through packing, we consistently observe that the short β‐strand at Gly_207_‐Asn_210_ occurs with the formation of the α‐helix at Gly_212_‐Val_218_, suggesting these structures are linked. In the β‐pseudo‐active structures, daughter helix α2 is pulled away from the ds‐DNA‐binding site, suggesting the ds‐DNA interaction is needed to position the daughter helix α2 and facilitate the formation of the β‐strand at Gly_207_‐Asn_210_. Thus, α‐ and β‐pseudo‐active states seem mutually exclusive, with the long N‐terminal helix acting like a spring to pull the α‐ and β‐pseudo‐active states apart, an effect that is removed when ds‐DNA binding breaks this helix (Fig. 2).

The α‐ and β‐pseudo‐active states observed here are therefore consistent with existing data and support the Civril et al. model for ds‐DNA activation. Interestingly, the 4KB6 structure shows Asp_227_ (Asp_202_ in *S. scrofa* numbering) interacting with Mg^2+^ and the α‐phosphate oxygens of ATP. Since the physiological concentration of ATP is ∼10‐fold its *K*_d_ or *K*_M_, these data suggest cGAS may be in the β‐pseudo‐active state in cells with the long N‐terminal helix broken. If so, the element missing for cGAS activation would be the induction and alignment of the short β‐strand at Gly_207_‐Asn_210_ and the α‐helix at Gly_212_‐Val_218_. We therefore propose efforts to stimulate the innate immune response through cGAS should focus on the discovery of binders that facilitate the formation of the short β‐strand at Gly_207_‐Asn_210_ and the α‐helix at Gly_212_‐Val_218_. Since we have always observed both the short β‐strand at Gly_207_‐Asn_210_ and the α‐helix at Gly_212_‐Val_218_ to occur simultaneously in our α–pseudo‐active states, it may be possible to induce both these structures through stabilizing the short β‐strand at Gly_207_‐Asn_210_ in the presence of high concentrations of ATP. We therefore envision a small molecule binder that could mimic the phosphate backbone interactions of ds‐DNA in aligning the end of the daughter helix α2 after the N‐terminal helix is broken, this should induce a short β‐strand at of Gly_207_‐Asn_210_, thus stimulating cGAS activation at high ATP concentrations.

In addition to structural studies, we have engaged in an analysis of the catalytic mechanism of cGAS. These studies include a novel SPR‐based enzymatic assay that should be applicable to other systems (Fig. 3). In these experiments, ds‐DNA bound cGAS did not show a direct RU effect during catalysis, necessitating the use of STING_155–341_ as a sensor protein for 2′,3′‐cGAMP. Although it is possible to use other binding sensors, such as antibodies tailored to detect specific analytes, a prior SPR‐based catalytic assay showed a non‐zero RU effect for an enzyme undergoing catalysis^29^; it is therefore reasonable to speculate a direct use of SPR for enzymology studies should be possible for other systems.

When compared with the NMR and HPLC assays used here, SPR has the advantage of being a continuous and relatively quick data collection method. Its disadvantages are that it cannot separate side reactions from the main reaction without associated sensor proteins (e.g. STING_155–341_ here), and it is hard to quantitate turnover rates. Furthermore, since SPR systems have a continuous flow, their use for enzymology must meet certain constraints: (1) as *K*_M_ is a steady‐state measurement, the analyte produced must reach steady‐state response (RU plateau) within the experiment; (2) the same flow rate must be used for all injections; and (3) the reaction cannot proceed exclusively through intermediates with weak affinities that will be lost to the flow.

cGAS produces AMP‐2′‐GTP as an intermediate to 2′,3′‐cGAMP. Full length cGAS experiments using NMR and HPLC clearly demonstrate that AMP‐2′‐GTP is a significant initial product of cGAS in a closed reaction vessel. These data are consistent with prior studies which also show linear dinucleotides are produced by cGAS^19^, ^20^; however, these data are distinct in that they are from continuous assays capable of probing early time points to distinguish the relative rates of formation of linear *v* cyclic dinucleotide. Furthermore, using SPR we demonstrate 2′,3′‐cGAMP can be produced through a process that is not competitive with ATP and GTP [Fig. 4(e)]. The simplest explanation of the NMR, HPLC and SPR data is that while the majority of AMP‐2′‐GTP product is released, a portion can instead reorient in the cGAS active site to produce 2′,3′‐cGAMP in a mechanism that is not competitive with ATP or GTP.

NMR and HPLC experiments using full length cGAS demonstrate AMP‐2′‐GTP or linear homo‐nucleotides are the major initial products at physiological concentrations of ATP and GTP [Fig. 4(d)]. To our knowledge no one has yet demonstrated the presence of AMP‐2′‐GTP in cells, though many have now demonstrated its presence *in vitro*. Our protein was produced without any post translational modifications, it is possible that such modifications, or multiple localization of cGAS upon long segments of ds‐DNA in cells,^30^ could lead to a lower fraction of linear nucleotides produced. Indeed, our SPR data could be thought of as a mimic for the ds‐DNA localized condition. However, lacking cell data, we can still say these HPLC and MS data show linear homo‐dinucleotides are readily produced at high (> 1 m*M*) concentrations of a single nucleotide and do not elicit an SPR binding response for STING_155–341_, suggesting STING_155–341_ does not bind AMP‐3′‐ATP or GMP‐2′‐GTP. Furthermore, neither apo cGAS_161_ nor STING_155–341_ binds AMP‐2′‐GTP at up to 50 μ*M*. Although weak AMP‐2′‐GTP binding may be dismissed as an artifact of cGAS truncation or the SPR system, these data are borne out by an apparent weak affinity for ds‐DNA‐bound full length cGAS in solution NMR experiments. That AMP‐2′‐GTP has relatively weak affinity for cGAS is supported by its steady‐state concentration of 250 μ*M* at physiologically relevant (∼10‐fold their *K*_M_) ATP and GTP concentrations [Fig. 4(a)].^27^ If the catalytic efficiency (*k*_cat_/*K*_M_) of AMP‐2′‐GTP formation and depletion are equivalent the intermediate would have a *K*_M_ around 25 μ*M*.

The apparent reconciliation of the weak affinity of AMP‐2′‐GTP with the importance of producing 2′,3′‐cGAMP for immune signaling is that STING is a strong binder of 2′,3′‐cGAMP, thus only a small amount of 2′,3′‐cGAMP is need to activate STING. SPR shows a portion of the cGAS_161_ reaction occurs without competition with ATP and GTP. If the portion of 2′,3′‐cGAMP produced through a non‐competitive mechanism is greater than 0.1% of the cellular concentration of ATP (∼ 2 m*M*), this minor portion will produce the requisite concentration of 2′,3′‐cGAMP needed for STING signaling before the AMP‐2′‐GTP released from cGAS can accumulate to sufficiently compete with ATP and GTP for enzyme rebinding.

Given their unusual 2′,5′‐phosphodiester bond, which would distinguish them from normal RNA‐like oligos, there is a possibility AMP‐2′‐GTP and GMP‐2′‐GTP have non‐STING binding partner. However, until such a partner is identified it would seem the large amount of linear homo‐nucleotides and AMP‐2′‐GTP released compared with 2′,3′‐cGAMP is part of a futile cycle for cGAS. In conclusion, given the low basal activity of cGAS,^18^, ^19^, ^31^ its apparent bias to not bind GTP, and therefore not produce the unusual 2′,5′‐phosphodiester bond, and that much of its activity seems to be lost in apparent futile cycles involving homo‐ or hetero‐dinucleotide release, this enzyme seems to contain several levels of control to limit 2′,3′‐cGAMP production in the absence of stimulatory ds‐DNA.

## Materials and Methods

### Protein expression

The genes for full length *H. sapiens* cGAS, N‐terminal truncated cGAS beginning at residue 161 (cGAS_161_), and *H. sapiens* STING residues 155 through 341 (STING_155–341_) were ordered from GeneWiz (South Plainfield, NJ). Genes were cloned into pET28 containing an N‐terminal SUMO‐HIS_6_ tag, a ULP1 cleavage site, a BIRA recognition sequence, and a TEV cleavage site.

*Escherichia coli* BL21(DE3) cells transformed with the above constructs were grown in LB medium (Invitrogen) at 37°C to an OD_600 nm_ of 0.8 before inducing protein expression with 0.1 m*M* isopropyl‐1‐thio‐β‐d‐galactopyranoside at 15°C for 16–20 h. Harvested cells were suspended in 20 m*M* HEPES pH 7.5, 1 *M* NaCl, 10% (v/v) glycerol, 30 m*M* imidazole and 1 m*M* TCEP, and gently killed using a Branson Ultrasonic Disintegrator (VWR Scientific Products, Chicago, IL) with seven rounds of 10 s 10% duty cycle sonication separated by 50 s rest periods.

The soluble fraction was separated using centrifugation (30,000 RCF, 1 h), applied to a HisTrap FF column (GE Healthcare), washed with 10 column volumes of buffer containing 20 m*M* HEPES pH 7.5, 250 m*M* NaCl, 10% glycerol, and 1 m*M* TCEP containing 30 m*M* imidazole, then eluted in the same buffer but with 300 m*M* imidazole and 300 m*M* NaCl. The protein was concentrated using a 10 kDa MWCO Amicon spin column (Millipore), buffer exchanged into 50 m*M* HEPES pH 7.5, 250 m*M* NaCl, 10% (v/v) glycerol and 1 m*M* TCEP. For SPR studies, cGAS_161_ or STING_155–341_ was treated with ULP1 and BirA ligase (Avidity) to generate N‐terminal biotin‐tagged protein; otherwise, samples were incubated for 16–20 h with TEV protease (Life Technologies) to liberate untagged protein. Protease‐treated samples were passed through a HisTrap FF column to remove tags and residual tagged protein. Full length cGAS or cGAS_161_ was applied to a Heparin FF column in 20 m*M* HEPES pH 7.5, 250 m*M* NaCl, 10% glycerol, and 1 m*M* TCEP, then eluted with a gradient of 0.25–1 *M* NaCl. All proteins were subjected to a final purification step using a HiLoad Superdex75 column (GE Healthcare) in 20 m*M* HEPES pH 7.5, 150 m*M* KCl and 1 m*M* TCEP. Protein purity was verified by SDS‐PAGE and ESI‐TOF mass spectrometry.

### Crystallization and structure determination

Dinucleotides were purchased (InvivoGen). Compounds F_1_, F_2_ and F_3_ were discovered as the result of an NMR‐based fragment screen (see Hall et al. for general description of library and methods^32^). Crystals of cGAS_161_ were grown using conditions similar to a previous report.^31^ Protein was concentrated to 6 mg/mL, and then mixed at a 2:1 ratio with PEG 3350 (18–20% v/v), 0.2 M ammonium citrate pH 7 in a sitting drop well at 4°C. Rod‐shaped crystals were observed within 2 days, and grew to their final size within 5–7 days. Cryoprotectant was made using mother liquor at a final concentration of 23% PEG 3350. Compounds were dissolved into cryoprotectant at 50 m*M*, and soaks were performed at 4°C for 5–10 min. Crystals were flash frozen in liquid nitrogen, and data were collected at the Argonne National Lab (IMCA) beamline. Data were scaled and merged using AIMLESS.^33^ Initial phases were obtained from MR using PDB 4LEV in PHASER.^34^ Refinement was performed using BUSTER‐TNT and Phenix Refine. Omit maps were calculated using Phenix Refine with simulated annealing.

### HPLC assays

GTP titrations experiments were performed using 500 n*M* full length cGAS, 1 μ*M* interferon stimulatory ds‐DNA (ISD) (Integrated DNA Technologies) in 10 m*M* HEPES pH 7.5, 140 m*M* NaCl, 5 m*M* MgCl_2_ and 0.01% Tween‐20 at 37°C. ATP was held at 1.1 m*M*, while GTP was titrated down from 1.1 m*M* to 80 μ*M* using a 60% dilution series. Reactions were quenched with 50 m*M* EDTA and separated on a Zorbax SB‐C8 column (5 μm, 4.6 × 150 mm) using a methanol‐phosphate gradient (buffer A: 20 m*M* potassium phosphate, pH 6.0; buffer B: equal volumes methanol and buffer A) at 35°C. Peaks were identified using ATP, GTP, and 2′,3′‐cGAMP chemical standards. 2′,3′‐cGAMP peak areas were converted to molar concentrations using a standard curve. Formation of this product was fit to a time‐dependent approach to steady‐state using equation (1):

(1) $$\left[P\right]=\;\frac{V_{1}-V_{2}}{k_{obs}}*(1-exp(-k_{obs}*t))+V_{2}*t$$

where *V*_1_ is the initial rate and was constrained to zero, *k*_obs_ is the rate constant for the approach to the steady state rate (*V*_2_), and *t* is time. Steady‐state values were analyzed for substrate‐dependent inhibition using equation (2):

(2) $$V_{obs}=\;\frac{V_{max}*\left[S\right]}{K_{M}+\left[S\right]\left(1+\frac{\left[S\right]}{K_{IS}}\right)}$$

where *V*_obs_ is the observed reaction velocity at substrate concentration *S, V*_max_ is the theoretical maximal rate, and *K*_M_ and *K*_IS_ are the apparent Michaelis constant and the apparent inhibition constant. Although titrating GTP, a second product peak was identified with an inverse dependence on the GTP concentration. Maximal rate of production was seen in the absence of GTP, suggesting the peak was the linear AMP‐3′‐ATP product reported by Gao *et al*.^19^ (Supporting Information Fig. S4). The GTP‐dependence for the rate of AMP‐3′‐ATP formation was fit to a standard half‐maximal inhibitory concentration (*IC_50_*) model, described in equation (3):

(3) $$V_{obs}=\;\frac{V_{0}}{1+[GTP]/{IC}_{50}}$$

where *V*_0_ is the observed rate in the absence of GTP. Additionally, cGAS was titrated with 0.3–3 m*M* ATP in the absence of GTP. The rate of AMP‐3′‐ATP formation showed a hyperbolic concentration dependence, and was fit to the Michaelis Menten equation.

### NMR assays

Samples for continuous monitoring of cGAS reactions were prepared with 0.5 μ*M* cGAS, a top concentration of 2 m*M* ATP with 2‐fold dilutions over three points, and a fixed concentration of 500 μ*M* GTP. Reactions were performed in SPR running buffer (see below) at 23°C. Reactions were monitored through 1D spectra collected at 8 min intervals. Peak identities were determined from comparison to ATP, GTP and 2′,3′‐cGAMP standards, the assignment of additional peaks as the linear AMP‐2′‐GTP intermediate was made after mass spectra analysis revealed a mass of 853 Da (predicted 853 Da) in addition to the substrates and product. Compound concentrations were determined through peak integration using ATP as an internal standard.

Steady‐state rates of 2′,3′‐cGAMP formation were fit using equation.(1) Initial rates of intermediate formation were determined by fitting the intermediate concentration to equation (1) with an unconstrained V_1_ value and a negative V_2_ value.

*K*_M_ values were determined using fixed ATP (690 μ*M*) and variable GTP concentrations (90, 180, 360, or 550 μ*M*) or fixed GTP (770 μ*M*) and variable ATP concentrations (70, 125, 300, or 500 μ*M*) at 37°C. Data were fit to the Michaelis Menten equation.

### MS assays

cGAS (100 n*M*) was incubated for 30 min at 37°C with 100 n*M* ISD in 10 m*M* HEPES pH 7.5, 140 m*M* NaCl, 5 m*M* MgCl_2_ and 0.01% Tween‐20, and varied substrate concentrations. The substrate dependence was assessed by titrating ATP or GTP over a range of 5 μ*M* to 1.3 m*M*, keeping the invariant substrate at 0.3 m*M* (GTP) or 1 m*M* (ATP). Additional samples were prepared using 2 m*M* ATP or GTP in the absence of the second nucleotide, or 0.5 m*M* GTP, 2 m*M* ATP. All samples were quenched with 50 m*M* EDTA prior to analysis. Quenched samples were diluted in H_2_O, vortexed, and centrifuged at 3000 RCF. Soluble analytes were separated in a hypercarb column (5 μm, 2 × 30 mm) using an acetate‐acetonitrile/acetone gradient (Buffer A: 20 m*M* ammonium acetate, pH 10; Buffer B: 45% acetonitrile, 45% acetone, 10 m*M* ammonium acetate, pH 10, 0.1% formic acid) at 60°C. Analytes were identified by mass spec analysis using a 1290 Agilent UHPLC in conjunction with a Sciex 5500 triple‐quadrupole mass spectrometer in MRM mode. Data were processed using Sciex's Multiquant 3.0. Reaction products were identified by mass and MRM transitions for each nucleotide: AMP‐3′‐ATP at 837 m/z (predicted and observed), GMP‐2′‐GTP at 869 m/z (predicted and observed), AMP‐2′‐GTP at 853 m/z (predicted and observed) and 2′3′‐cGAMP at 675 m/z (predicted and observed). ATP, GTP, and 2′3′‐cGAMP quantities were calculated from standard curves of peak intensity. Data were analyzed using equation (2).

### SPR binding assays

SPR experiments were performed using CM5 sensor chips in a BIACORE T200 (GE Healthcare). CM5 channels were functionalized with neutravidin (Pierce) through amine coupling (GE Healthcare) at 25°C to a density of 25,000–30,000 RUs. N‐terminal biotinylated cGAS_161_, STING_155–341_, or 3′‐biotinylated ISD were immobilization at 5 μL/min, 4°C. No protein was added to Channels 1 and 2 held apo cGAS_161_ (final density ∼7000 RU), Channel 3 held a 1:1 molar equivalence of ISD:cGAS_161_ (final density ∼6000 RU), and Channel 4 held STING_155–341_ (final density ∼10,000 RU).

Two‐fold dilution series of compounds F_1_‐F_3_ (300–0.3 μ*M*) or cyclic dinucleotides (500–0.5 μ*M*) were prepared in 20 m*M* HEPES pH 7.5, 150 m*M* KCl, 20 m*M* MgCl_2_ and 1 m*M* TCEP, 1% DMSO. Samples were injected for 30–60 s, and dissociation was measured for 30–60 s at 60 μL/min, 4°C.

Sample‐dependent responses on Channels 2–4 were subtracted from Channel 1 to account for interactions with neutravidin, and data were further corrected by subtracting a zero concentration blank from all compounds to account for mismatches in the sample buffer and running buffer. Dissociation equilibrium constants (*K*_d_) were determined using a binary association model (T200 BIA Eval software):

(4) $$K_{d}=\frac{C*R_{max}}{R_{C}-R_{0}}-C$$

where *R*_C_ is the response at compound concentration *C*, *R*_max_ is the maximum response of the fit, and *R_0_* is a global data offset from zero (Table 1).

### SPR activity assays

The ATP‐concentration dependence of cGAS activity was assessed by injecting ATP (2.0 m*M* to 2.0 μ*M*, 2‐fold dilutions) in the presence of 1.0 m*M* to 1.0 μ*M* GTP (2‐fold dilutions). ATP and GTP titrations were also performed in the absence of the other nucleotide. 2′,3′‐cGAMP (20 μ*M* to 20 n*M*, 2‐fold dilutions) was injected before and after ATP and GTP samples to establish the 2′,3′‐cGAMP‐dependent change on the STING channel.

SPR instrumentation and sensor chip setup was as described earlier. Samples were injected for 900 s, dissociation was measured for 300 s at 5 μL/min, 4°C. The STING response (Channel 4) values showed an initial negative deflection, especially at higher nucleotide concentrations; therefore, blank subtracted values were normalized to have zero absorbance at the signal minimum (∼100 s). Normalized data were fit to a single exponential association from 600 to 900 s, and the extrapolated plateau RU values were then replotted as a function of the ATP concentration. Datasets corresponding to different GTP concentrations were simultaneously fit to equation (2) using GraphPad Prism with a shared *K*_M.ATP_ value. In turn, the resulting *V*_max_ values were fit as a function of the GTP concentration using the Michaelis Menten equation (equation 2 when [S]/*K*_IS_ ≪1).

To further probe the cGAS substrate inhibition observed above, SPR injections were made using a 2:1 molar ratio of ATP to GTP over an ATP range of 5 m*M* to 90 μ*M* (11 points, 1.5‐fold dilutions). Data collection and analysis were as described earlier.

To ensure that STING_155–341_ response was specific to 2′,3′‐cGAMP and not influence by any of the linear products present in the enzymatic samples (e.g. the cGAS intermediate), reactions were prepared in the buffer used for SPR with 2 μ*M* cGAS, 3 μ*M* ISD, and a 2:1 molar ratio of GTP to ATP at 0.25, 0.5, 1.0, and 4 m*M* ATP. Reactions were quenched with 85 m*M* EDTA after 3 h at 23°C and analyzed by NMR to determine the concentration of 2′,3′‐cGAMP and linear 2′,5′‐GMP‐3′‐AMP dinucleotide. The samples were then diluted 4‐fold into SPR running buffer to minimize noise associated with running buffer and sample buffer mismatch and injected for 60 s, and dissociation was measured for 60 s at 60 μL/min, 4°C.

## Author contributions

All authors contributed to the intellectual development of these works. J.H. and E.R. wrote the article. J.H., L.B., and E.R. created illustrations. S.S. and J.H. produced protein. J.H. and L.B. performed structural studies. J.H. and E.R. developed the SPR assay, J.H. performed SPR assays. H.W. developed the NMR assays, H.W. and R.H. performed NMR assays. E.R. performed HPLC assays and kinetic analysis. A.B. and D.D. performed MS assays.

## Supporting information

Supplementary Figure Legends

Supporting information Figure 1

Supporting information Figure 2

Supporting information Figure 3

Supporting information Figure 4

Supporting information Table 1

Supporting information Table 2

Supporting information Table 3
