# Supplementary material for: The catalytic mechanism of cyclic GMP‐AMP synthase (cGAS) and implications for innate immunity and inhibition
Source: Protein Sci. 2017 Oct 25;26(12):2367–80. doi: 10.1002/pro.3304 (PMC5699495; doi:10.1002/pro.3304)
Supplement: Supplementary file 1 — Supplementary Figure Legends [file PRO-26-2367-s001.docx]

SUPPLEMENTARY FIGURE LEGENDS

**Fig. S1. Compounds bound to the cGAS active site.** a) 2ʹ,2ʹ-cGAMP, b) 2ʹ,3ʹ-cGAMP, c) 3ʹ,3ʹ-cGAMP, d) 3ʹ,3ʹ-cdIMP, e) 3ʹ,3ʹ-cdUMP, f) 2ʹ,5ʹ-GpAp, compounds g) F_1_, h) F_2_, and i) F_3_. Compounds structures are shown above protein structures; Fo-Fc density (green) is shown contoured at 3 Sigma; F_2_ and F_3_ are modeled with more than a single pose due to uncertainty arising from ambiguous electron density and internal pseudo-symmetry. Figure generated using Pymol with pdb structures 5VDO (cGAS161●2′,2′-cGAMP), 5VDP (cGAS161●2′,3′-cGAMP), 5VDT (cGAS161●3′,3′-cGAMP), 5VDR (cGAS161●3′,3′-cdIMP), 5VDS (cGAS161●3′,3′-cdUMP), 5VDQ (cGAS161●2′,5′-GpAp), 5VDW (cGAS161●F_1_), 5VDU (cGAS161●F_2_), and 5VDV (cGAS161●F_3_).

**Fig. S2. Compound binding sensorgrams.** 2ʹ,3ʹ-cGAMP binding to a) apo cGAS_161_ (89 ± 6 μM), and b) ds-DNA bound cGAS_161_ (56 ± 1 μM). 3ʹ,3ʹ-cGAMP binding to c) apo cGAS_161_ (21 ± 5 μM), and d) ds-DNA bound cGAS_161_ (8.9 ± 0.3 μM). Apo cGAS_161_ binding to e) ATP (235 ± 97 μM), compounds f) F_1_ (236 ± 19 μM), g) F_2_ (64 ± 3 μM), and h) F_3_ (80 ± 4 μM). Binding fits are inset in each sensorgram, colors in fit points correspond to the same colors in the sensorgrams. Compounds F_1_ and F_2_ have extra site binding at concentrations above 100 μM. Data are the average and standard deviation of two or more experiments.

**Fig. S3. Ile_220_ and Ser_221_ do not form a beta-turn conformation in our 2ʹ,3ʹ-cGAMP structure.** a) Overlay of our structure of cGAS in complex with 2ʹ,3ʹ-cGAMP (5VDP ) and 4O67 (blue) showing the difference in modeling of Gly_207_-Ser_221_. Sequence shown below with residues missing from our structure underlined b) View of the beta-turn showing the difference in the modeled position of Val_218_ in the two models. c) A 180° rotation of panel b with electron density (blue mesh) shows a close approach of Lys_219_ and Ala_222_, making the direction of the main chain ambiguous at this position. The side chain clearly defines the registry of Tyr_215_ and Val_218_ in our structure, indicating I_220_ and S_221_ are disordered and do not form a beta-turn in our structure. 2Fo-Fc density is shown contoured at 1.5 Sigma, figure generated using Pymol.

**Fig. S4. cGAS produces a linear AMP-3ʹ-ATP, GMP-2ʹ-GTP, and AMP-2ʹ-GTP *in vitro* under reaction conditions mimicking physiological nucleotide concentrations.** a) Extracted Ion Chromatographs (EIC) at 5 ppm error are shown for the 3 dinucleotide intermediates, ATP, GTP, and 2ʹ,3ʹ-cGAMP produced using at 2 mM ATP, 0.5 mM GTP as start conditions. Single nucleotide reactions (ATP or GTP) produced only homo-nucleotide products. b) The corresponding MS2 fragmentation mass spectrums are shown for each metabolite. Data was acquired on a Q-Exactive Plus with a 140,000 hz resolution for MS1. Subsequent MS2 data was collected using 70,000 hz (MS1) and 35,000 hz (MS2) resolution.
