# Supplementary figures and images for: The catalytic mechanism of cyclic GMP‐AMP synthase (cGAS) and implications for innate immunity and inhibition
Source: Protein Sci. 2017 Oct 25;26(12):2367–80. doi: 10.1002/pro.3304 (PMC5699495; doi:10.1002/pro.3304)

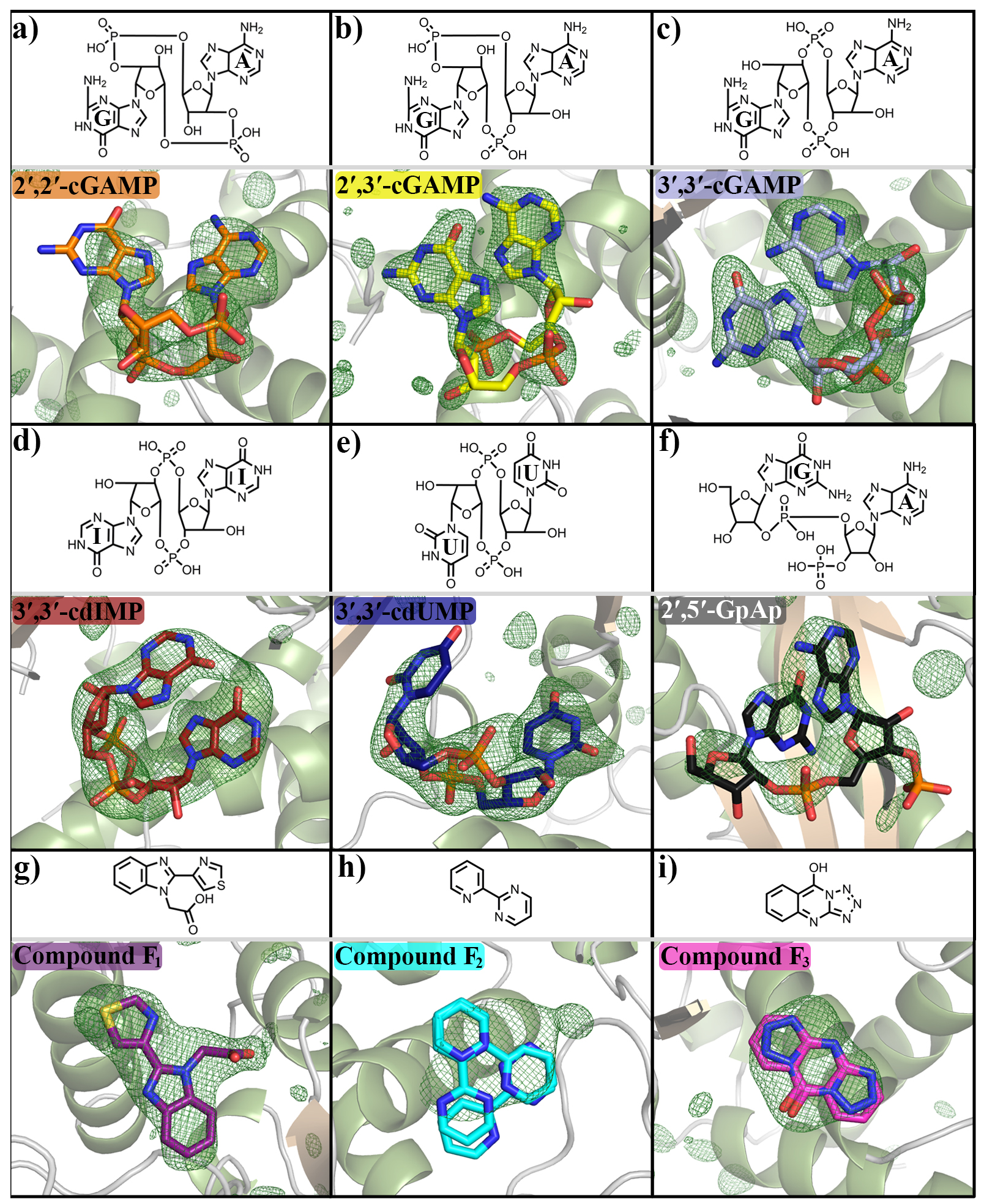

Supplement: Supplementary file 2 — Supporting information Figure 1 [file PRO-26-2367-s002.tiff]

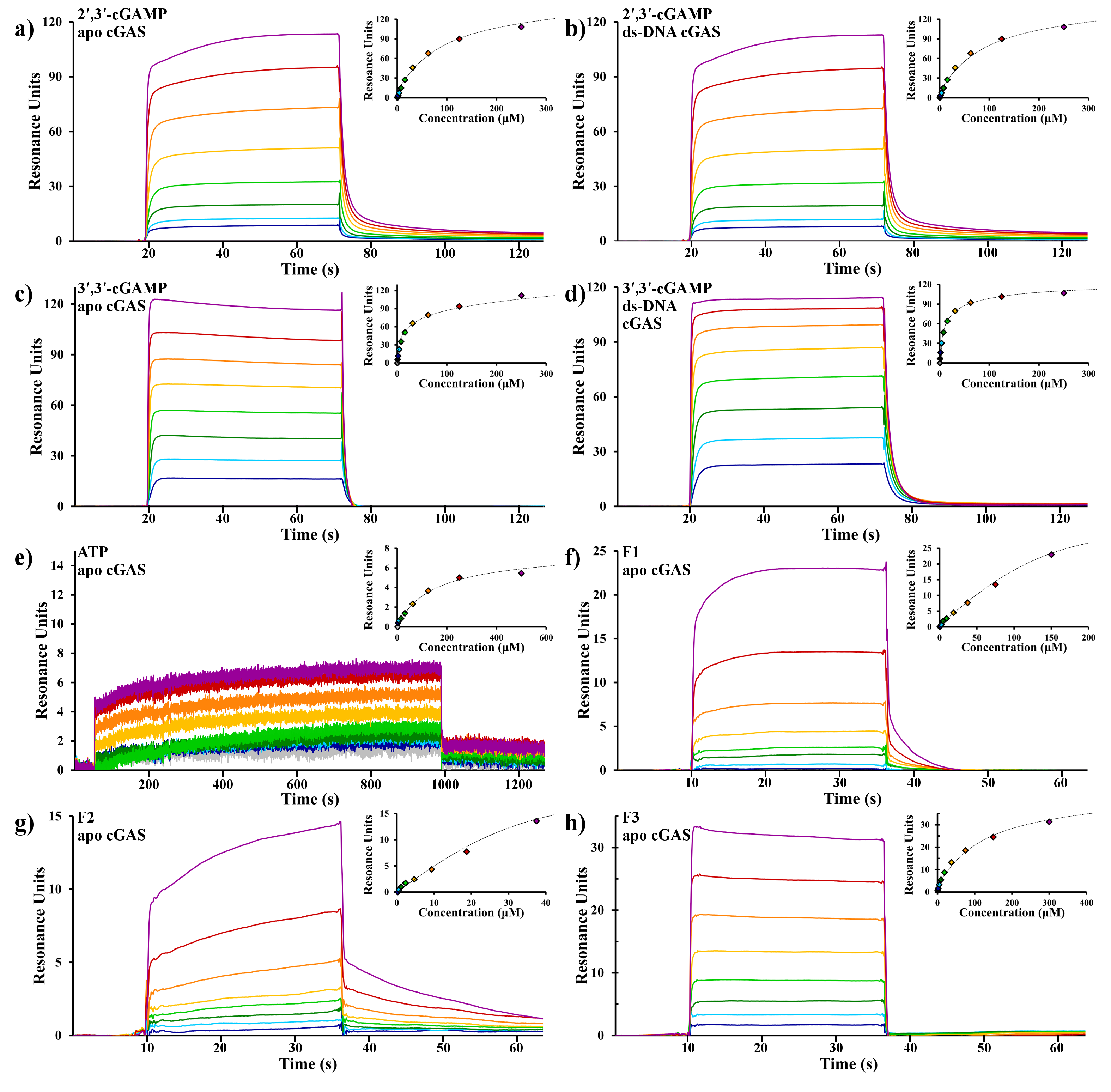

Supplement: Supplementary file 3 — Supporting information Figure 2 [file PRO-26-2367-s003.tiff]

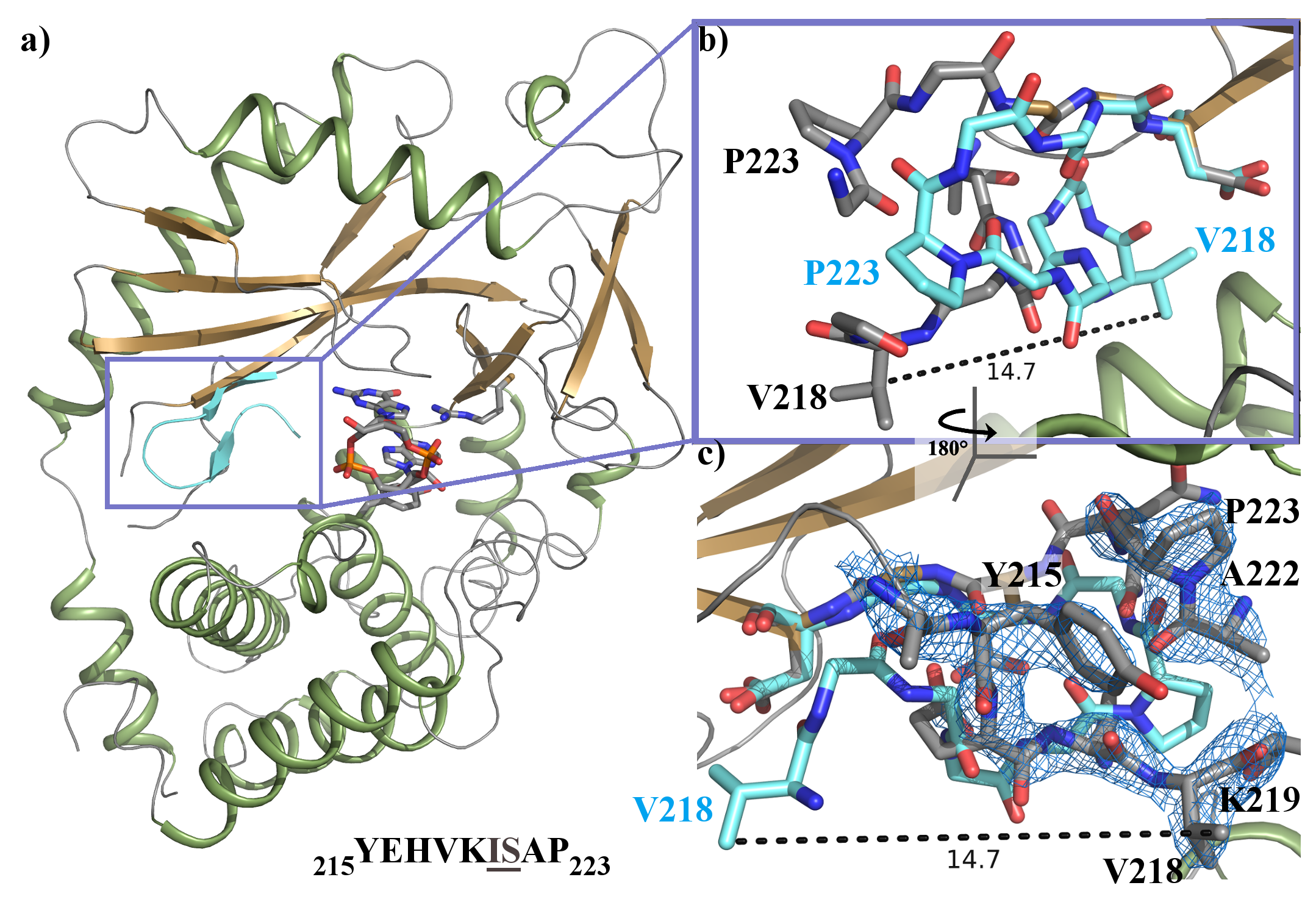

Supplement: Supplementary file 4 — Supporting information Figure 3 [file PRO-26-2367-s004.tiff]

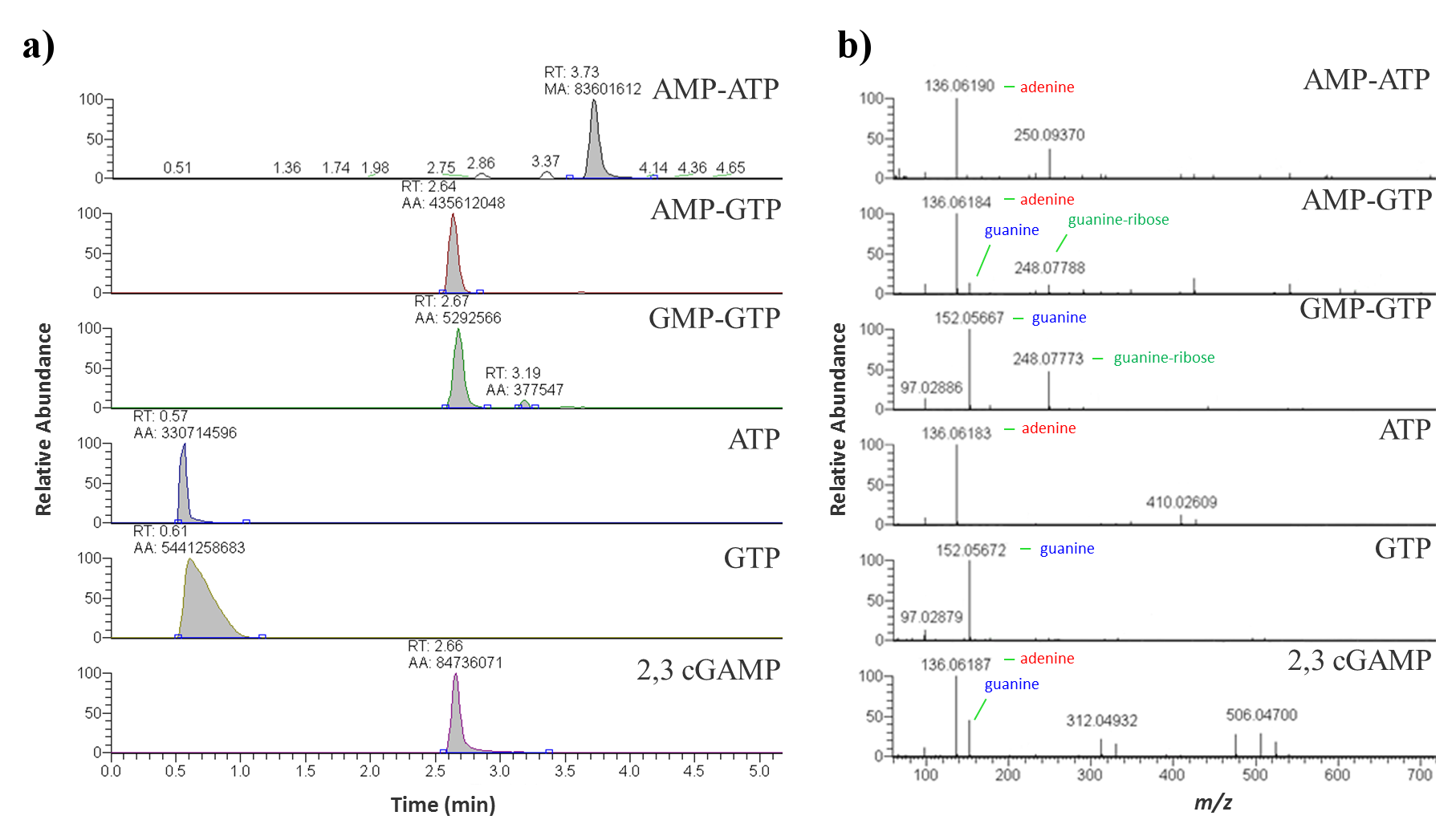

Supplement: Supplementary file 5 — Supporting information Figure 4 [file PRO-26-2367-s005.tiff]
