# Supplementary material for: The catalytic mechanism of cyclic GMP‐AMP synthase (cGAS) and implications for innate immunity and inhibition
Source: Protein Sci. 2017 Oct 25;26(12):2367–80. doi: 10.1002/pro.3304 (PMC5699495; doi:10.1002/pro.3304)
Supplement: Supplementary file 6 — Supporting information Table 1 [file PRO-26-2367-s006.docx]

**SI Table 1. Crystallographic data and refinement statistics for cGAMP structures**

| PDB Code | cGAS_161_  2′,2′-cGAMP  5VDO | cGAS_161_  2′,3′-cGAMP  5VDP | cGAS_161_  3′,3′-cGAMP  5VDT |
| --- | --- | --- | --- |
| X-ray source | APS (IMCA) | APS (IMCA) | APS (IMCA) |
| Wavelength (Å) | 1.00 | 1.00 | 1.00 |
| Space group | C2 | C2 | C2 |
| Unit cell |  |  |  |
| a, b, c (Å) | 216.4, 47.67, 88.86 | 215.9, 46.73, 88.17 | 213.9, 47.97, 88.27 |
| α, β, γ (°) | 90.00, 110.2, 90.00 | 90.00, 110.5, 90.00 | 90.00, 110.1, 90.00 |
| Resolution(Å)^a^ | 83.4-3.22 (3.39-3.22) | 50.0-2.33 (2.41-2.33) | 82.9-2.58 (2.72-2.58) |
| No. of reflections |  |  |  |
| Total | 75,523 (7,064) | 131,749 (6,949) | 185,232 (12,754) |
| Unique | 14,176 (2,030) | 37,195 (1,790) | 26,726 (3,861) |
| Completeness (%) | 99.8 (99.5) | 100 (96.8) | 98.7 (99.5) |
| I/σ(I) | 16.1 (3.6) | 9.30 (1.85) | 14.8 (2.2) |
| R_meas_ (%)  CC_1/2_ (%) | 19.9 (52.3)  99.0 (85.5) | 12.9 (89.5)  99.6 (77.9) | 21.3 (77.1)  98.4 (69.7) |
| **Refinement** | |  |  |
| Resolution (Å)^a^ | 55.7-3.22 (3.47-3.22) | 32.45-2.30 (2.36-2.30) | 55.3-2.58 (2.67-2.58) |
| No. of reflections used | 14,157 (2,770) | 35,983 (1,958) | 26,697 (2,663) |
| R_work_/R_free_ (%) | 20.5 (27.7)/25.8 (37.8) | 19.7 (29.4)/24.3 (34.1) | 20.2 (30.5)/23.6 (34.6) |
| rms deviations |  |  |  |
| Bond length (Å) | 0.002 | 0.007 | 0.003 |
| Bond angles (°) | 0.577 | 1.004 | 0.647 |
| No. of atoms | 5,783 | 6,044 | 5,931 |
| Protein | 5,689 | 5,754 | 5,693 |
| Ligands | 92 | 161 | 92 |
| Water | 2 | 129 | 146 |
| Ave. B-factors (Å^2^) |  |  |  |
| Protein | 104.5 | 47.10 | 65.20 |
| Ligand | 128.1 | 54.60 | 44.30 |
| Water | 78.60 | 38.50 | 46.60 |
| Ramachandran (%) |  |  |  |
| Favored  Allowed | 95.2  4.8 | 96.6  3.1 | 95.9  4.1 |
| Outliers | 0 | 0.3 | 0 |

(a) Values in brackets are for the highest resolution bin.
