# Supplementary material for: The catalytic mechanism of cyclic GMP‐AMP synthase (cGAS) and implications for innate immunity and inhibition
Source: Protein Sci. 2017 Oct 25;26(12):2367–80. doi: 10.1002/pro.3304 (PMC5699495; doi:10.1002/pro.3304)
Supplement: Supplementary file 7 — Supporting information Table 2 [file PRO-26-2367-s007.docx]

**SI Table 2. Crystallographic data and refinement statistics for non-cGAMP dinucleotide structures**

| PDB Code | cGAS_161_  3′,3′-cdIMP  5VDR | cGAS_161_  3′,3′-cdUMP  5VDS | cGAS_161_  2′,5′-GpAp  5VDQ |
| --- | --- | --- | --- |
| X-ray source | APS (IMCA) | APS (IMCA) | APS (IMCA) |
| Wavelength (Å) | 1.00 | 1.00 | 1.00 |
| Space group | C2 | C2 | C2 |
| Unit cell |  |  |  |
| a, b, c (Å) | 215.5, 48.40, 89.10 | 215.5, 48.41, 86.09 | 215.2, 47.63, 88.37 |
| α, β, γ (°) | 90.00, 109.9, 90.00 | 90.00, 104.8, 90.00 | 90.00, 110.1, 90.00 |
| Resolution(Å)^a^ | 101-3.04 (3.21-3.04) | 104-2.77 (2.92-2.77) | 101-3.25 (3.42-3.25) |
| No. of reflections |  |  |  |
| Total | 54,411 (8,541) | 197,956 (18,447) | 44,783 (6,446) |
| Unique | 16,476 (2,447) | 22,387 (3,224) | 13,619 (1,973) |
| Completeness (%) | 96.9 (99.7) | 100.0 (99.9) | 99.5 (99.8) |
| I/σ(I) | 11.1 (2.2) | 10.8 (2.2) | 11.1 (2.1) |
| R_meas_ (%)  CC_1/2_ (%) | 13.9 (67.6)  99.3 (81.7) | 26.4 (92.3)  95.6 (70.0) | 12.2 (69.8)  99.2 (99.2) |
| **Refinement** | |  |  |
| Resolution (Å)^a^ | 55.9-3.04 (3.28-3.04) | 58.2-2.77 (2.89-2.77) | 55.5-3.25 (3.50-3.25) |
| No. of reflections used | 16,476 (3,354) | 22,373 (2,763) | 13,599 (2,685) |
| R_work_/R_free_ (%) | 20.8 (28.4)/25.4 (32.2) | 19.7 (26.5)/24.3 (30.9) | 20.8 (30.4)/26.5 (38.7) |
| rms deviations |  |  |  |
| Bond length (Å) | 0.002 | 0.003 | 0.003 |
| Bond angles (°) | 0.604 | 0.581 | 0.677 |
| No. of atoms | 5,851 | 5,698 | 5,773 |
| Protein | 5,643 | 5,526 | 5,679 |
| Ligands | 176 | 82 | 94 |
| Water | 32 | 90 | 0 |
| Ave. B-factors (Å^2^) |  |  |  |
| Protein | 83.90 | 53.00 | 90.40 |
| Ligand | 102.3 | 58.50 | 112.8 |
| Water | 48.50 | 30.80 | NA |
| Ramachandran (%) |  |  |  |
| Favored  Allowed | 96.6  3.4 | 95.5  4.5 | 95.6  4.26 |
| Outliers | 0 | 0 | 0.14 |

(a) Values in brackets are for the highest resolution bin.
