# Supplementary material for: The catalytic mechanism of cyclic GMP‐AMP synthase (cGAS) and implications for innate immunity and inhibition
Source: Protein Sci. 2017 Oct 25;26(12):2367–80. doi: 10.1002/pro.3304 (PMC5699495; doi:10.1002/pro.3304)
Supplement: Supplementary file 8 — Supporting information Table 3 [file PRO-26-2367-s008.docx]

**SI Table 3. Crystallographic data and refinement statistics for fragment structures**

| PDB Code | cGAS_161_  Compound F_1_  5VDW | | cGAS_161_  Compound F_2_  5VDU | cGAS_161_  Compound F_3_  5VDV | |
| --- | --- | --- | --- | --- | --- |
| X-ray source | APS (IMCA) | | APS (IMCA) | APS (IMCA) | |
| Wavelength (Å) | 1.00 | | 1.00 | 1.00 | |
| Space group | C2 | | C2 | C2 | |
| Unit cell |  | |  |  | |
| a, b, c (Å) | 217.4, 48.00, 86.86 | | 216.4, 48.02, 86.47 | 217.6, 46.63, 89.70 | |
| α, β, γ (°) | 90.00, 105.3, 90.00 | | 90.00, 104.9, 90.00 | 90.00, 111.4, 90.00 | |
| Resolution(Å)^a^ | 75.9-2.71 (2.86-2.71) | | 83.5-2.73 (2.88-2.73) | 83.5-3.00 (4.24-3.00) | |
| No. of reflections |  | |  |  | |
| Total | 74,765 (11,515) | | 74,570 (11,493) | 171,956 (110,116) | |
| Unique | 23,537 (3,433) | | 22,940 (3,365) | 17,370 (11,114) | |
| Completeness (%) | 98.3 (99.0) | | 98.5 (99.6) | 100 (100) | |
| I/σ(I) | 10.3 (2.70) | | 10.8 (2.30) | 11.0 (5.80) | |
| R_meas_ (%)  CC_1/2_ (%) | 18.3 (90.2)  98.0 (70.5) | | 13.9 (83.7)  99.1 (69.6) | 18.9 (37.0)  99.5 (75.9) | |
| **Refinement** | |  | | |  |
| Resolution (Å)^a^ | 52.4-2.71 (2.83-2.71) | | 52.3-2.73 (2.85-2.73) | 55.3-3.00 (3.19-3.00) | |
| No. of reflections used | 23,517 (2,939) | | 22,914 (2,894) | 17,350 (2,847) | |
| R_work_/R_free_ (%) | 22.2 (31.5)/25.7 (35.4) | | 22.2 (32.5)/25.2 (36.7) | 20.5 (27.5)/25.7 (34.8) | |
| rms deviations |  | |  |  | |
| Bond length (Å) | 0.003 | | 0.002 | 0.002 | |
| Bond angles (°) | 0.714 | | 0.665 | 0.468 | |
| No. of atoms | 5,639 | | 5,734 | 5,492 | |
| Protein | 5,565 | | 5,587 | 5,324 | |
| Ligands | 38 | | 98 | 102 | |
| Water | 36 | | 52 | 66 | |
| Ave. B-factors (Å^2^) |  | |  |  | |
| Protein | 61.60 | | 65.30 | 89.70 | |
| Ligand | 71.30 | | 80.80 | 87.80 | |
| Water | 30.60 | | 37.30 | 73.30 | |
| Ramachandran (%) |  | |  |  | |
| Favored  Allowed | 96.5  3.5 | | 96.1  3.90 | 94.5  5.5 | |
| Outliers | 0 | | 0 | 0 | |

(a) Values in brackets are for the highest resolution bin.
